# Catalytically Active Carbon From Cattail Fibers for Electrochemical Reduction Reaction

**DOI:** 10.3389/fchem.2019.00786

**Published:** 2019-11-19

**Authors:** Yanyan Liu, Meifang Hu, Wei Xu, Xianli Wu, Jianchun Jiang

**Affiliations:** ^1^Institute of Chemical Industry of Forest Products, Chinese Academy of Forestry, Nanjing, China; ^2^Key Laboratory of Biomass Energy and Material of Jiangsu Province, Nanjing, China; ^3^College of Chemistry, Zhengzhou University, Zhengzhou, China

**Keywords:** nitrogen-doped carbon, hierarchical pores, biomass pyrolysis, hydrogen evolution reaction (HER), oxygen reduction reaction (ORR), catalytic active carbon

## Abstract

Catalytically active carbons derived from plant biomass are conducive to the construction of renewable energy source system and utilization of sustainable resources. In this article, natural cattail fibers are used to fabricate porous nitrogen-doped carbon via direct chemical activation and heteroatom modification treatments. The graphene-like sheets from biomass pyrolysis are assembled into three-dimensional carbon frameworks. The chemical activation of KHCO_3_ generated unique porous structure and N-containing molecules pyrolysis modification provided nitrogen doping atoms. High surface area up to 2,345 m^2^·*g*^−1^ with simultaneous hierarchical pores (from micro to meso and macro) with abundant edge defects are achieved for these carbon materials. These materials have a very large external surface area up to 1,773 m^2^·*g*^−1^. The above strategy exhibits a significant synergistic effect on the improvement of catalytic properties toward hydrogen evolution reaction and oxygen reduction reaction. The small over potentials and Tafel slopes of these catalytically active carbons demonstrate excellent potential applications in renewable energy conversion and storage systems. This research established a new link among environmental improvement, biomass conversion and renewable energy utilization.

## Introduction

The carbon cycle in nature provides us with a useful resource and energy warehouse. The useful content should be taken out from the carbon cycle based on plant biomass for human society development. The harvest of renewable energy, extraction of chemical resource, and fabrication of functional material from bulk biomass is a promising research direction. It is one of the important industrial ways for plant biomass conversion to produce commercial activated carbon with adsorption properties and supports for heterogeneous catalyst active components (Deng et al., 2016; Fan et al., 2018; Gao et al., 2018). It is interesting to note that the catalytic active material obtained from biomass will be beneficial to the formation of a perfect self-consistent cycle system. The elemental components in plant biomass, especially the structural uniqueness, provide a rich development space for the design of efficient active catalysts for renewable energy conversion and storage (Yu et al., 2016; Long et al., 2017; Chen et al., 2018). Nanocarbon catalysts with atomic-molecular level adjustable structures, excellent electronic conductivity, tailorable physicochemical properties and robust mechanical-chemical stability, exhibit promising potential of building an efficient catalytic system (Liu et al., 2018; Sun et al., 2018).

Several electrochemical processes, such as the hydrogen evolution reaction (HER) and oxygen reduction reaction (ORR), are of importance for the building of efficiently harvest, storage, and conversion system for renewable energy (Huang et al., 2018; Xiao et al., 2018; Yang et al., 2019). The sluggish kinetics of these electrochemical processes in overall water splitting, fuel cells and rechargeable metal-air batteries, are main problem for their extensive commercial reach. A critical strategy to boost the electrocatalytic activity is to adjust their chemical compositions and structures of carbon catalysts at atomic-molecular level. The pristine carbon materials lack appropriate adsorption capability or enough catalytic active sites for reactants and products. It is necessary to expand the exposure number of catalytic active sites and improve the intrinsic catalytic activity of carbon materials via the catalytic active site engineering and defect engineering (Gu et al., 2018; Hao et al., 2018; Ou et al., 2018; Zhuang et al., 2018; Jin et al., 2019). The intrinsic activity, number of active sites and exposure of efficient active sites on catalyst surface can be improved significantly by suitably surface adjusting and modification strategy. Heteroatom doping will disturb the electronic distribution state of catalyst surface, increase the electronic conductivity, adjust the adsorption energy barrier and enhance the intrinsic catalytic activity of materials (Ishii et al., 2017; Wang et al., 2017a; Fu et al., 2019; Zhou et al., 2019). The nitrogen doping has been recognized as an effective strategy for carbon catalysts to improve their intrinsic activity toward electro catalytic reactions (Singh et al., 2017; Li et al., 2018; Wu et al., 2018). The construction and adjusting of pore structure can enhance the exposure of active sites in carbon catalysts and improve the utilization efficiency of active sites via promote the diffusion and adsorption processes (Wang et al., 2017b; Xu et al., 2018). The edge defects also can be constructed via the effective chemical activation approach (Hao et al., 2018). The above strategy should be adopted together to promisingly optimize the structure and performances of biomass-derived carbon catalysts. In terms of the production route, those traditional manufactures of activated carbon are generally involved some pretreatment including hydrothermal or roasting, followed by chemical modification. Complex operation steps lead high energy consumption and economic costs.

In this article, we are trying to provide a new link or important branch in the construction of a plant biomass-based energy resource system based on carbon cycle. The plant biomass derived catalytically active carbon (CAC) is used as a platform to regulate and amplify the catalytic sites via direct chemical activation-carbonization and heteroatom-doping modification strategy. CAC is defined as carbon materials consisting of only non-metallic elements with somewhat activity of catalytic chemical reaction. The new idea is confirmed by the excellent catalytic performances of the hydrogen evolution reaction (HER) and oxygen reduction reaction (ORR), two important reaction processes for the renewable energy harvesting, fuel cells and metal-air batteries. It is feasible to use the CAC-based on plant biomass branch for energy storage and conversion. This research will be beneficial to reduce human society's dependence on traditional non-renewable fossil fuels.

## Experimental Section

### Preparation of Materials

Cattail fibers (2.55 g) were sufficiently ground with KHCO_3_ (10.20 g) to mix uniformly. Then the mixture was calcinated at 900°C for 2 h with a heating ramp of 5°*C*·*min*^−1^ in N_2_ atmosphere. After being cooled to room temperature naturally, the sample was slowly added to HCl solution (150 mL, 18.5 wt%) and stirred for 6 h at room temperature. After being filtered and washed with water, the hierarchically porous carbon fibers were obtained and denoted as HPCF. The HPCF (0.1 g) were pretreated in HNO_3_ (65 wt%, 30 mL) at room temperature for 40 min, washed with water and dried at 100°C. The treated sample (0.09 g) were added into hot melamine solution (0.333 g, 33 mL) and stirred for 10 min to disperse uniformly. Then the mixture was transferred into a Teflon-lined steel autoclave (50 mL), heated to 100°C and held for 320 min. After being filtered and dried, the compound was annealed at 400°C for 2 h, followed by heating to 550°C and kept for another 3 h with a heating rate of 2.3°*C*·*min*^−1^ in N_2_ atmosphere. After being cooled naturally, the N-doped hierarchically porous carbon fibers were generated and denoted as NHPCF. NCF was prepared as contrast sample via a similar procedure of NHPCF without the addition of KHCO_3_.

### Characterization

The morphologies of the as-prepared materials were studied by using transmission electron microscope (TEM, FEI Tecnai G^2^ F20 S-TWIN electron microscope, operating at 200 kV), combined with energy dispersive X-ray spectroscopy (EDS) and scanning electron microscopy (SEM, Zeiss Ultra 55). The atom force micro analysis was conducted on a MFP-3D Infinity Asylum Research AFM (AFM, Oxford Instruments). The phase structures of products were characterized with X–ray diffraction (XRD, Bruker D8 advance with Cu Kα, λ = 1.5418 Å). Raman spectra were recorded on a Renishaw RM−1000 with Ar-ion laser (λ = 514 nm, power = 50 mW). Fourier transform infrared (FT-IR) spectra were carried out by using a Nicolet 380 spectrometer. X-ray photoelectron spectra (XPS) were executed by applying a PHI quantera SXM spectrometer (Al excitation source, K_α_ = 1486.60 eV), where binding energies were calibrated by referencing the C1s peak (284.8 eV) to reduce the sample charge effect. The N_2_ sorption isotherms were measured by employing surface area and poresize analyzer (ASAP2420-4MP, Micromeritics, USA) at 77 K. From the adsorption branch of isotherm curves in the P/P° range of 0.05–0.35, the specific surface areas (*S*_BET_) of samples were calculated by the multi-point Brunauer–Emmett–Teller (BET) method. The *t*-plot method was used to calculate the microporous volume (*V*_micro_), microporous area (*S*_micro_), and exteranal area (*S*_external_). The single point total pore volume (*V*_total_) was determined from the amount adsorbed at the relative pressure of about 0.999. The pore size distribution was evaluated by the non-localized density function theory (NLDFT) method. Elemental analysis (EA) of samples was operated by using the elementar vario (Thermo Flash EA 1112).

### Electrochemical Measurements

Electro catalytic hydrogen evolution reaction(HER) and oxygen reduction reaction (ORR) measurements were performed in a three-electrode system at 25°C, and the data were recorded using a CHI 760E electrochemistry workstation coupled with a rotating ring-disk electrode (RRDE) system (Pine Instruments Co. Ltd., USA). The reference electrode for HER and ORR were a saturated calomel electrode (SCE) and a Hg/HgO electrode, respectively. The counter electrodes for HER and ORR were a graphite rod and a platinum wire, respectively. The potentials for HER and ORR were referenced to reversible hydrogen electrode (RHE) by following calculations (1) and (2), respectively:

(1) E(vs.RHE) = E(vs.SCE) + 0.241 + 0.059pH

(2) E(vs.RHE) = E(vs.Hg/HgO) + 0.098 + 0.059pH

In order to obtain catalytic ink, catalysts (3 mg) were put into mixed solution containing ethanol (500 μL) and nafion (0.5 wt%, 50 μL). Then the mixture was sonicated for 40 min to obtain a homogeneous ink. The dispersion (15 μL, including 82 μg catalyst) was transferred onto a polished glassy carbon electrode (5 mm diameter), and dried at room temperature. The glassy carbon electrodes well-proportioned coated with samples (loading amount of 0.417 mg·*cm*^−2^) were used as the working electrode. Linear sweep voltammetry (LSV) was conducted in H_2_-saturated 0.5 M H_2_*SO*_4_ for HER and O_2_-saturated 0.1 M KOH for ORR at a scan rate of 5 mV·*s*^−1^. The presented current density was normalized to the geometric surface area of electrodes. Electrochemical impedance spectroscopy (EIS) was carried out at 1,600 rpm in the frequency region of 0.025–100,000 Hz. The poison tests for ORR were performed in mixed solution containing KOH (0.1 M) and CH_3_OH (1 M). Rotating ring disk electrode (RRDE) measurements were carried out in O_2_-saturated KOH (0.1 M) at 1,600 rpm with a scan rate of 5 mV·*s*^−1^, and the potential of the Pt ring was set at 1.5 V (vs RHE). The electron transfer number (n) and the yield of hydrogen peroxide released during ORR were calculated based on the following equation:

(3) $$n=4\times \frac{I_{D}}{I_{D}+I_{R}/N}$$

(4) $$\left[{\text{H}}_{2}{\text{O}}_{2}\right]\%=200\times \frac{I_{R}/N}{I_{R}/N+I_{D}}$$

Where *I*_D_ is the disk current, *I*_R_ is the ring current, and *N* is the collection coefficient of the Pt ring (*N* = 0.37). Commercial Pt/C catalysts were used as a reference to evaluate the electrocatalytic performance of various samples. CV cycles with different scan rates were measured during the potential window to estimate the double layer capacitance (*C*_dl_) and then to calculate the electrochemically active surface area (ECSA). The working electrode was held at each potential vertex for 10 s before beginning the next sweep. The ECSA were determined according to the following equations:

(5) $$ECSA=C_{\text{dl}}/C_{\text{s}}$$

Where C_s_ is the specific capacitance value for a flat standard with 1 cm^2^ of real surface area. The general value of C_s_ is 20.9 μ*F*·*cm*^−2^ for the ECSA calculation of carbon materials.

## Results and Discussion

**Figure 1:**
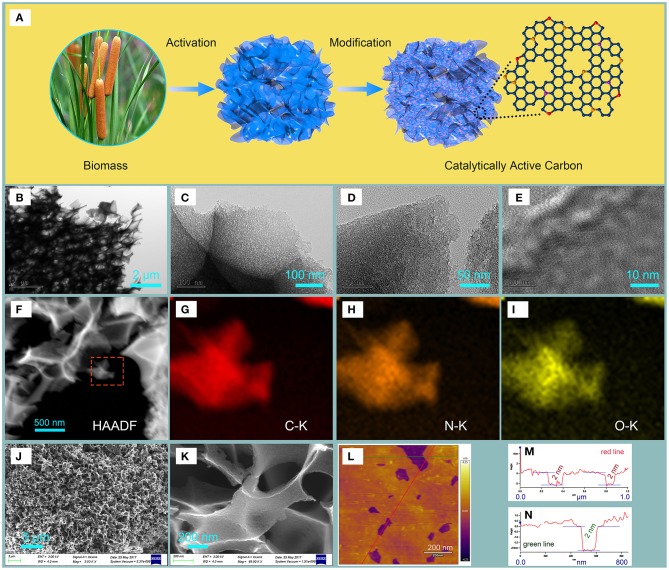
The **(A)** Preparation of NHPCF from cattail spikes, **(B–E)** TEM images, **(F)** HAADF images, **(G–I)** EDX elemental mapping images of NHPCF, **(J,K)** SEM image, **(L)** AFM image, and **(M,N)** the corresponding height profiles of NHPCF.

A two-step method is designed and used to prepare NHPCF. The formation steps of NHPCF are illustrated in Figure 1A. Firstly, KHCO_3_ is used as activator to promote the carbonization of cattail spike. This step is obviously different from those previous reporting works, in which pre-carbonization or hydrothermal treatment often is necessary. The main possible reactions are proposed to revolve around some redox steps (Experimental section for details). In these steps, mesopores and micropores are produced by the reaction between activator and as-formed carbon intermediate. The chemical reaction between carbon and activator generates more micropore. The formation of CO_2_ gas expands carbon to create mesopore. Macropores are generated by the expansion of gas products. Thus, the carbon materials (HPCF) possessing hierarchical pores are obtained during the chemical activation process. Then, the nitrogen source is introduced to HPCF for the formation of N-doped porous carbon. Finally, NHPCF possessing hierarchical porous structure and N elements is acquired successfully. No toxic volatile organic solvents and dangerous reagents are used throughout the preparation process.

The morphology and structure of the samples are investigated by SEM, TEM and AFM analysis. The SEM images of cattail fibers displayed tubular morphology with smooth surface (Figures S1a,b). No pore can be observed in the structure of cattail fibers. NCF showed similar morphology to that of cattail fibers, demonstrating that N-doping modification and carbonization treatment had little effect on the structure of material (Figure S1c). It could be seen that NCF exhibited sheet-like structure, and there is no obvious surface pore (Figure S1d). Significantly, HPCF showed three-dimensional carbon frameworks network structure with macropores after KHCO_3_ activation at 900°C (Figure S1e). Porous carbon sheets are confirmed via TEM image of HPCF, which indicated the important effect of KHCO_3_ on the structural transformation of materials (Figure S1f). After N-doping modification, NHPCF presented analogous 3D network structure and porous carbon sheets to that of HPCF (Figures 1B–E). Porous structure is beneficial for the increasing of the surface areas of materials and fully exposure of edge-defective and in-plane active sites for catalytic reaction. The EDX elemental mappings obviously showed that the C, O and N elements of NHPCF are homogeneously distributed in the framework (Figures 1F–I). These EDX images and EDX spectrum image (Figure S2) proved that N elements are successfully doped into carbon framework of NHPCF. The 3D network structure with macropores of NHPCF is confirmed by SEM images (Figures 1J,K). The sheet-thickness of NHPCF is probed by AFM analysis (Figures 1M,N). The images revealed that some macropore structure existed in NHPCF (Figure 1L), which is consistent with SEM images. The line scan profiles showed an average layer thickness of 2 nm for NHPCF (Figures 1M,N). The average thickness is in accordance with the thickness of other reported carbon materials (Wu et al., 2009; Shen et al., 2013; Amiinu et al., 2016). Porous carbon thin sheets stacked up to generate a lot of defects. This stack structure may be useful for the improvement of their potential catalytic activity.

**Figure 2:**
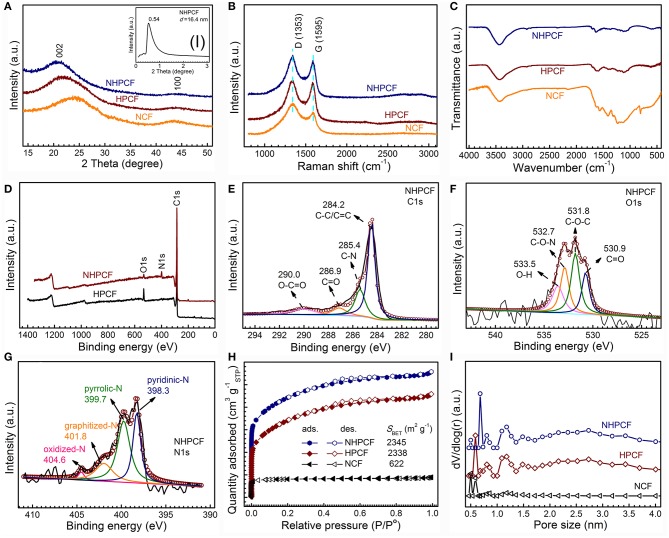
**(A)** XRD patterns of NCF, HPCF, and NHPCF (inset shows small angle XRD of NHPCF), **(B)** Raman spectra, **(C)** FT-IR spectra of NCF, HPCF, and NHPCF, **(D)** XPS survey spectra of HPCF, and NHPCF, **(E–G)** C1s spectra, O1s spectra, and N1s spectra of NHPCF, **(H)** Nitrogen adsorption-desorption isotherms and **(I)** pore size distributions calculated by NLDFT method of NCF, HPCF, and NHPCF.

The XRD patterns of NCF, HPCF, and NHPCF are displayed in Figure 2A. It could be seen that they exhibited similar diffraction peaks. The two broad peaks at about 24.5° and 43.5° are ascribed to the (002) and (100) lattice plane of carbon phase, respectively (JCPDS, No.75-1621) (Xie et al., 2016). The results suggested the formation of most amorphous or disordered carbon in samples. The characteristic peak of NHPCF moved a little to the left, indicating gradually increasing lattice of graphene sheets. This may be caused by to the wrinkle structure of nitrogen doped carbon. In addition, small-angle diffraction pattern revealed that NHPCF presents good layer structure (the inset in Figure 2A). According to the Bragg equation, the interlayer spacing is 16.4 nm. The interlayer spacing from XRD patterns is completely in accordance with the lamellar thickness from TEM observation. Raman spectra are also collected to further confirm the graphitic degree of samples (Figure 2B). It is observed that all the samples displayed similar spectrum. The D bands centered at 1,353 cm^−1^ are attributed to the disordered/defective structure of carbon; while the G bands centered at 1,588 cm^−1^ are due to the graphitic sp^2^ carbon (Sheng et al., 2011; Zhou et al., 2016). The *I*_D_/*I*_G_ values are 1.19, 1.04, and 1.14 for NCF, HPCF, and NHPCF, respectively. Compared with HPCF, the higher *I*_D_/*I*_G_ value of NHPCF showed that carbon confusion degree increased and generated more structural defects after N-doping modification treatment. It often is beneficial for the boosting of electrochemical catalytic activity (Liang et al., 2015). FT-IR spectra are collected to qualitatively study the chemical functional groups of samples (Figure 2C). The peaks at 3,435 cm^−1^ for all samples are owing to the O–H stretching vibration (adsorbed water or hydroxyl groups) (Zhao et al., 2015). The peaks at 1,241–1,636 cm^−1^ of NCF are typical aromatic heterocyclic C-N stretching vibration (Tian et al., 2014). The peak near 806 cm^−1^ of NCF derived from C_3_N_4_ fragment (Peng et al., 2016). Compared with NCF, it can be clearly seen that some peaks of HPCF and NHPCF diminish or disappear. This phenomenon is due to dehydration or corrosion function during the KHCO_3_ activation process. For NHPCF, the peak near 1,635 cm^−1^ is ascribed to C=O stretching vibration (Gao et al., 2017). The peak around 1,458 cm^−1^ is due to C=C or C=N bands (Zhang et al., 2015). The broad peaks centered at 1,116 cm^−1^ are the characteristic of C–O bonds (Gao et al., 2017). The results revealed that N elements are doped successfully into the framework of NHPCF, which may have a positive impact in improving the electrochemical performance (Gao et al., 2017).

XPS measurements are carried out to further probe the surface chemical compositions of samples. The XPS survey spectrum revealed the existence of C, O and N elements in NHPCF (Figure 2D). The C1s spectrum of NHPCF can be deconvoluted into four peaks, ascribing to the sp^2^-hybridized graphitic carbon C-C/C=C (284.2 eV), C-N (285.4 eV), C=O (286.9 eV) and O-C=O (290.0 eV), respectively (Figure 2E) (Guo et al., 2015). The O1s spectrum of NHPCF could be fitted into four peaks which are due to C=O (530.9 eV), C-O-C (531.8 eV), C-O-N (532.7 eV), and O-H (533.5 eV), respectively (Figure 2F) (Perazzolo et al., 2015). The N1s spectrum of NHPCF displayed four characteristic peaks, which are attributed to pyridinic-N (398.3 eV), pyrrolic-N (399.7 eV), graphitized-N (401.4 eV), and oxidized N (403.5 eV), respectively (Figure 2G) (Sun et al., 2017). These results further proved that *N* elements are successfully doped into the carbon material. Pyridine-N is conductive to fast charge-transfer rate and the high surface polarity that had significant effects in the electrocatalytic processes. Based on elemental analysis, the atomic percentage of *N* elements for HPCF, NHPCF, and NCF are 0.49, 6.35, and 5.48%, respectively (Table S1). The *N* ratios from XPS spectra are 0.62 and 7.43% for HPCF and NHPCF, respectively (Table S2). The results indicated that the N element is obviously doped into the NHPCF through hydrothermal and calcinations process.

The porosities of NCF, HPCF and NHPCF are further researched by N_2_ adsorption-desorption isotherms. Typical mixture of I and IV adsorption isotherm curves with E hysteresis loop according to the IUPAC classification, indicate the coexistence of micropores and mesopores (Figure 2H). The specific surface area of NCF, HPCF, and NHPCF are 622, 2,338, and 2,345 m^2^·g^−1^, respectively. The micro area and external surface area are 555 and 67, 626, and 1,712 m^2^·g^−1^, 572 and 1,773 m^2^·g^−1^ for NCF, HPCF, and NHPCF, respectively (Table S3). The increased ratio of external surface area indicated that the activation of KHCO_3_ played an important role in the formation of mesopores during pyrolysis process. After N-doping modification, NHPCF showed decreased micro area and increased external surface area compared with HPCF. The results revealed that N elements are doped into micropores and generated defects to produce mesopores. In addition, the pore size distribution curves also proved the hierarchically porous structure. The pore size distribution curves of samples via NLDFT method show the pore size ranging from 0.4 to 4.0 nm (Figure 2I). The micropores may be generated by the escape of gas during the reaction process. Due to the chemical activation of KHCO_3_ and N-doping modification, NHPCF possesses large surface area, hierarchically porous structures and more structural defects. These textual features are beneficial to the exposure of more active sites and facilitate mass transport and electron transfer during the electrocatalytic process (Liu et al., 2016).

**Figure 3:**
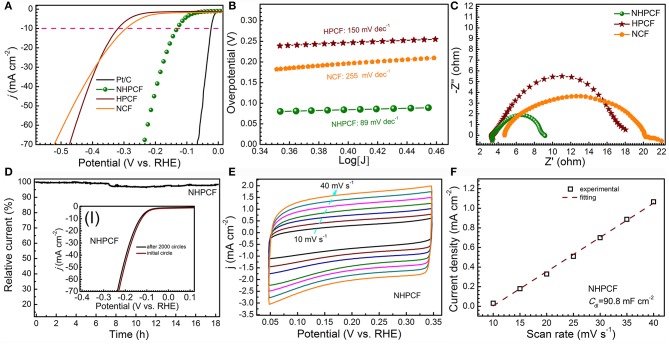
**(A)** LSV curves for Pt/C (10%), NCF, HPCF, and NHPCF in 0.5 M H_2_SO_4_ solution with scan rate of 5 mV·s^−1^, **(B)** Tafel plots **(C)** Nyquist plots of NCF, HPCF, and NHPCF, **(D)** current–time plot at −0.15 V (vs. RHE) (inset shows initial polarization curves for NHPCF and that after 2,000 cycles of potential sweeps), **(E)** CV curves of NHPCF at different scan rates, and **(F)** corresponding evaluation of the C_dl_ for NHPCF.

LSV curves for samples are firstly measured in 0.5 M H_2_SO_4_ solution with scan rate of 5 mV·s^−1^ (Figure 3A). Unquestionably, Pt/C exhibited the highest activity with smallest overpotentials. NHPCF electrode displayed small onset overpotential of 80 mV and needed low overpotential of 150 mV to afford current density of 10 mA·cm^−2^. HPCF and NCF showed higher overpotentials of 330 mV and 300 mV at 10 mA·cm^−2^, respectively. The HER activity of samples is also evaluated through Tafel plots (Figure 3B). The measured value for NCF, HPCF and NHPCF is 255, 150, and 89 mV·dec^−1^, respectively. The Tafel slope hinted that a Volmer-Heyrovsky mechanism may be involved for HER on this carbon electrode (Oh et al., 2016). The low Tafel slope and small overpotential of NHPCF suggested its excellent catalytic performances. These results demonstrated that the HER activity of NHPCF is significantly enhanced through chemical-activation and N-doping modification treatments (Zhang et al., 2016a). Electrochemical impedance spectroscopy (EIS) measurements of all samples are carried out to explore the charge transfer kinetics during HER processes (Figure 3C). Compared with NCF and HPCF, NHPCF had smallest charge transfer resistance (*R*_ct_) of 5.91 Ω. The lower *R*_ct_ means the faster electron transfer rate in electrochemical process. In addition, the chronoamperometry test is recorded at −0.15 V (vs. RHE) to evaluate the electrochemical stability of NHPCF. NHPCF exhibited high current retention over 90% after long-term cycles for 18 h (Figure 3D). After the continuous CVs at 100 mV·s^−1^ for 2,000 cycles, the LSV curves of NHPCF had a negligible shift of 0.007 V to reach a current density of 10 mA·cm^−2^ (the inset I in Figure 3D). The above results illustrated that NHPCF had good catalytic stability for HER. ECSA is considered to be a useful parameter to reflect the exposure degree of the active sites of electro catalysts (Zuo et al., 2017). The C_dl_ of NCF, HPCF and NHPCF are calculated from CV curves with different sweep speeds in 0.5 M H_2_SO_4_ solution (Figure 3E and Figures S3a,c) (Benck et al., 2012). The CV curves for these samples are close to rectangular shape, indicating good conductivity and strong ability of the electrolyte transport. The *C*_dl_ of NHPCF is obtained to be 90.79 mF·cm^−2^ by calculating the slope from the fitted line (Figure 3F), which is close to that of NCF (100 mF·cm^−2^) and HPCF (98.83 mF·cm^−2^) (Figures S3b,d). Therefore, the ECSA calculated from *C*_dl_ of NCF, HPCF and NHPCF are 478.5, 472.9, and 434.4 m^2^·g^−1^, which are close to each other. Due to the chemical activation and N-doping modification, more electrochemical active sites with high intrinsic activity of NHPCF electrode are utilized during the electro catalytic process. These results are consistent with the catalytic properties of various samples.

**Figure 4:**
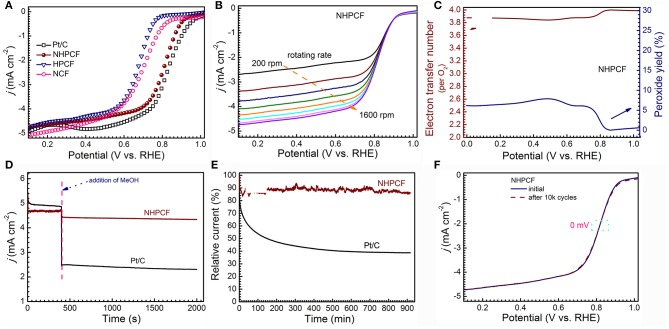
**(A)** LSV curves of NCF, HPCF, NHPCF and Pt/C in O_2_-saturated 0.1 M KOH at 1,600 rpm with 5 mV·s^−1^, **(B)** LSV curves of NHPCF with different rotate speed in O_2_-saturated 0.1 M KOH, **(C)** the electron transfer number (*n*) and peroxide yield of NHPCF in O_2_-saturated 0.1 M KOH at 1,600 rpm, **(D)** Tolerance tests of NHPCF and Pt/C in O_2_-saturated KOH (0.1 M) with methanol (1 M) at 1,600 rpm, **(E)** Chronoamperometry curves of NHPCF and Pt/C electrodes at 0.45 V (vs. RHE) at 1,600 rpm and **(F)** Accelerated durability test (ADT) of NHPCF in O_2_-saturated 0.1 M KOH.

CV measurements are firstly performed to learn the ORR catalytic activity of samples (Figures S4a–c). All of catalysts showed obvious cathodic ORR peaks in O_2_-saturated KOH solution (0.1 M), but no any in N_2_-saturated KOH (0.1 M). Compared with NCF and HPCF, NHPCF presented a more positive peak potential, indicating its higher catalytic activity. To further study the ORR electrocatalytic process, LSV curves of NCF, HPCF, NHPCF, and Pt/C (20%) are tested by rotating disk electrode (RDE) in O_2_-saturated KOH solution (0.1 M) at 1,600 rpm (Figure 4A). NHPCF exhibited preferable activity toward ORR among these catalysts. The onset potential and the half-wave potential of NHPCF are 0.92 and 0.82 V, respectively, which are close to that of Pt/C. Because of the overcoming of diffusion limitation, NHPCF provided increasing reduction current density at higher rotation speeds (Figure 4B). In order to study the ORR reaction mechanism of NHPCF, the RRDE voltammograms are measured to evaluate the electron transfer number (n) and H_2_O_2_ yields (Figure S4d). The average n value is calculated to be 3.9, corresponding to a low H_2_O_2_ yield of 4.9 mol% (Figure 4C). These results revealed a dominant 4e^−^ reduction pathway in the ORR process (Li et al., 2017). Therefore, NHPCF is a quite promising candidate for the noble-metal based ORR catalysts. The oxidations of CH_3_OH on Pt-based catalysts are serious impediments for the stable electrochemical performances in fuel cells. Thus, the poisoning effects of CH_3_OH toward NHPCF are measured through i–t chronoamperometric response in O_2_-saturated KOH solution (0.1 M) with CH_3_OH (1 M). Compared with Pt/C (20%) catalysts, NHPCF showed a tiny current density loss, declaring excellent tolerance to the CH_3_OH poisoning effect (Figure 4D). The stability of catalyst is one of the major concerns in current alkaline fuel cell technology. The stability of Pt/C and NHPCF also is tested by using i–t chronoamperometric response at 0.45 V (Figure 4E). As a comparison, NHPCF displayed the highest stability, maintaining a high relative current of 88.2% after 54,000 s, whereas the commercial Pt/C (20%) catalysts revealed a low relative current of 43% under the same conditions. Additionally, accelerated degradation test (ADT) is carried out in O_2_-saturated KOH (0.1 M) by continuous CV tests between 0.2 and 1.2 V (vs. RHE) for 10,000 cycles at a scan rate of 100 mV·s^−1^. After ADT test, the half-wave potential (E_1/2_) for NHPCF shifted without clear deviation (almost 0 mV), further demonstrating its superior robust stability (Figure 4F).

**Figure 5:**
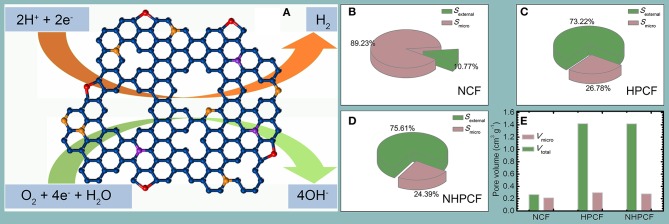
The **(A)** proposed catalytic mechanism of NHPCF CAC for HER and ORR, **(B–D)** surface areas composition, and **(E)** pore volumes of NCF, HPCF, and NHPCF.

Overall, the catalytic properties of NHPCF compare favorably to the value obtained using most other carbon-based catalysts (Tables S4, S5). The superior HER and ORR activity of NHPCF could be rationalized as follows: the pyridinic-N species in carbon framework provide more in-plane active sites for catalytic reaction (Zhang et al., 2016b). The hierarchical pores are conductive to the exposure of edge-defective active sites and facilitate sufficient mass-transport of reactants and products (Zhan et al., 2018). Higher external surface area guarantees the availability of more active sites during electrocatalytic process (Zeng et al., 2016). The three dimensional carbon framework configuration (hierarchical layer-like structure) of NHPCF offer loose textures and open spaces for easy diffusion and adsorption of electrolyte and efficient use of active sites (Figure 5A) (Jiang et al., 2017). The main achievement of this study is the successful regulation of micropore and mesoporous distribution. The proportion of external surface area in total surface area significantly increased (Figures 5B–D). In the modified CAC samples, the external surface becomes the main component. The ratio of total pore volume to microporous volume also increases significantly due to the increasing external surface area (Figure 5E). These changes in morphology can be considered as a reason for the improved apparent catalytic performances of CAC.

## Conclusions

In conclusion, highly efficient CAC material can be synthesized by using natural cattail fibers as raw materials via direct chemical activation and following N-doping modification treatments. Preferable HER performances, including early onset potentials, small Tafel slopes and excellent stability are obtained in acidic media with NHPCF as catalyst. NHPCF also presented high ORR activity, superior stability and tolerance to methanol poisoning effects in the alkaline media. The efficient catalytic performances of NHPCF toward HER and ORR should be assigned to the construction of unique hierarchical porous structure, high electrochemical active surface, defective carbon and sufficient N elemental doping structure. These positive results demonstrated that KHCO_3_ activation combined with N-doping modification is a promising method to create CAC materials from the original biomass for the diversity of potential applications. This work demonstrates a new design strategy for the conversion of renewable biomass into available electrochemical catalysts, which is beneficial to promote the sustainable development of society based on renewable energy harvest and conversion.

## Data Availability Statement

All datasets generated for this study are included in the article/Supplementary Material.

## Author Contributions

YL and JJ conceived and designed the experiments. YL and MH conducted the synthesis and measurements. YL, WX, and XW performed the structure analyses. YL wrote the manuscript. All authors attended the general discussion in the preparation process of this paper.

### Conflict of Interest

The authors declare that the research was conducted in the absence of any commercial or financial relationships that could be construed as a potential conflict of interest.

## References

[B1] Amiinu I. S. Zhang J. Kou Z. K. Liu X. B. Asare O. K. Zhou H.. (2016). Self-organized 3D porous graphene dual-doped with biomass sponsored nitrogen and sulfur for oxygen reduction and evolution. ACS Appl. Mater. Interfaces 8, 29408–29418. 10.1021/acsami.6b08719 27740758

[B2] Benck J. D. Chen Z. B. Kuritzky L. Y. Forman A. J. Jaramillo T. F. (2012). Amorphous molybdenum sulfide catalysts for electrochemical hydrogen production: insights into the origin of their catalytic activity. ACS Catal. 2, 1916–1923. 10.1021/cs300451q

[B3] Chen Y. M. Ji S. Wang H. Linkov V. Wang R. F. (2018). Synthesis of porous nitrogen and sulfur Co-doped carbon beehive in a high-melting-point molten salt medium for improved catalytic activity toward oxygen reduction reaction. Int. J. Hydrogen Energy 43, 5124–5132. 10.1016/j.ijhydene.2018.01.095

[B4] Deng J. Li M. M. Wang Y. (2016). Biomass-derived carbon: synthesis and applications in energy storage and conversion. Green Chem. 18, 4824–4854. 10.1039/C6GC01172A

[B5] Fan R. Y. Chen C. Han M. M. Gong W. B. Zhang H. M. Zhang Y. X.. (2018). Highly dispersed copper nanoparticles supported on activated carbon as an efficient catalyst for selective reduction of vanillin. Small 14:1801953. 10.1002/smll.201801953 30058300

[B6] Fu G. T. Wang Y. Tang Y. W. Zhou K. Goodenough J. B. Lee J.-M. (2019). Superior oxygen electrocatalysis on nickel indium thiospinels for rechargeable Zn–Air batteries. ACS Materials Lett. 1, 123–131. 10.1021/acsmaterialslett.9b00093

[B7] Gao L. Li C. T. Zhang J. Du X. Y. Li S. H. Zeng J. W. (2018). Simultaneous removal of NO and Hg^0^ from simulated flue gas over CoO_x_-CeO_2_ loaded biomass activated carbon derived from maize straw at low temperatures. Chem. Eng. J. 342, 339–349. 10.1016/j.cej.2018.02.100

[B8] Gao S. Y. Li X. G. Li L. Y. Wei X. J. (2017). A versatile biomass derived carbon material for oxygen reduction reaction, supercapacitors and oil/water separation. Nano Energy 33, 334–342. 10.1016/j.nanoen.2017.01.045

[B9] Gu Y. Chen S. Ren J. Jia Y. A. Chen C. M. Komarneni S.. (2018). Electronic structure tuning in Ni_3_FeN/r-GO aerogel toward bifunctional electrocatalyst for overall water splitting. ACS Nano 12, 245–253. 10.1021/acsnano.7b05971 29257880

[B10] Guo C. Z. Liao W. L. Li Z. B. Chen C. G. (2015). Exploration of the catalytically active site structures of animal biomass-modified on cheap carbon nanospheres for oxygen reduction reaction with high activity, stability and methanol-tolerant performance in alkaline medium. Carbon 85, 279–288. 10.1016/j.carbon.2015.01.007

[B11] Hao Y. J. Zhang X. Yang Q. F. Chen K. Guo J. Zhou D. Y. (2018). Highly porous defective carbons derived from seaweed biomass as efficient electrocatalysts for oxygen reduction in both alkaline and acidic media. Carbon 137, 93–103. 10.1016/j.carbon.2018.05.007

[B12] Huang Y. Wu D. F. Cao D. P. Cheng D. J. (2018). Facile preparation of biomass-derived bifunctional electrocatalysts for oxygen reduction and evolution reactions. Int. J. Hydrogen Energy 43, 8611–8622. 10.1016/j.ijhydene.2018.03.136

[B13] Ishii T. Maie T. Kimura N. Kobori Y. Imashiro Y. Ozaki J.-I. (2017). Enhanced catalytic activity of nanoshell carbon co-doped with boron and nitrogen in the oxygen reduction reaction. Int J Hydrogen Energy 42, 15489–15496. 10.1016/j.ijhydene.2017.05.003

[B14] Jiang H. Wang Y. Q. Hao J. Y. Liu Y. S. Li W. Z. Li J. (2017). N and P co-functionalized three-dimensional porous carbon networks as efficient metal-free electrocatalysts for oxygen reduction reaction. Carbon 122, 64–73. 10.1016/j.carbon.2017.06.043

[B15] Jin W. Chen J. P. Liu B. Hu J. G. Wu Z. X. Cai W. Q.. (2019). Oxygen vacancy–rich in-doped CoO/CoP heterostructure as an effective air cathode for rechargeable Zn–Air batteries. Small 15:1904210. 10.1002/smll.201904210 31559688

[B16] Li J. M. Wang W. Wang F. X. Kang Y. M. Tan T. Lei Z. Q. (2018). Astragali radix-derived nitrogen-doped porous carbon: an efficient electrocatalyst for the oxygen reduction reaction. Int J Hydrogen Energy 43, 551–561. 10.1016/j.ijhydene.2018.10.052

[B17] Li X. Yao Y. Liu J. Zou Z. (2017). Highly microporous nitrogen doped graphene-like carbon material as an efficient fuel cell catalyst. Int J Hydrogen Energy 42, 19903–19912. 10.1016/j.ijhydene.2017.06.017

[B18] Liang H. W. Wu Z. Y. Chen L. F. Li C. Yu S. H. (2015). Bacterial cellulose derived nitrogen-doped carbon nanofiber aerogel: an efficient metal-free oxygen reduction electrocatalyst for zinc-air battery. Nano Energy 11, 366–376. 10.1016/j.nanoen.2014.11.008

[B19] Liu M. R. Hong Q. L. Li Q. H. Du Y. H. Zhang H. X. Chen S. M. (2018). Cobalt boron imidazolate framework derived cobalt nanoparticles encapsulated in b/n codoped nanocarbon as effcient bifunctional electrocatalysts for overall water splitting. Adv. Funct. Mater. 28:1801136 10.1002/adfm.201801136

[B20] Liu S. W. Zhang H. M. Zhao Q. Zhang X. Liu R. R. Ge X. (2016). Metal-organic framework derived nitrogen-doped porous carbon@graphene sandwich-like structured composites as bifunctional electrocatalysts for oxygen reduction and evolution reactions. Carbon 106, 74–83. 10.1016/j.carbon.2016.05.021

[B21] Long W. Y. Fang B. Z. Ignaszak A. Wu Z. Z. Wang Y. J. Wilkinson D. (2017). Biomass-derived nanostructured carbons and their composites as anode materials for lithium ion batteries. Chem. Soc. Rev. 46, 7176–7190. 10.1039/C6CS00639F 29075713

[B22] Oh S. Kim H. Kwon Y. Kim M. Cho E. Kwon H. (2016). Porous Co-P foam as an efficient bifunctional electrocatalyst for hydrogen and oxygen evolution reactions. J. Mater. Chem. A 4, 18272–18277. 10.1039/C6TA06761A

[B23] Ou G. Fan P. X. Ke X. X. Xu Y. S. Huang K. Wei H. H. (2018). Defective molybdenum sulfide quantum dots as highly active hydrogen evolution electrocatalysts. Nano Res. 11, 751–761. 10.1007/s12274-017-1684-2

[B24] Peng Z. Yang S. W. Jia D. S. Da P. M. He P. Al-Enini A. M. (2016). Homologous metal-free electrocatalysts grown on three-dimensional carbon networks for overall water splitting in acidic and alkaline media. J. Mater. Chem. A 4, 12878–12883. 10.1039/C6TA04426C

[B25] Perazzolo V. Durante C. Pilot R. Paduano A. Zheng J. Rizzi G. A. (2015). Nitrogen and sulfur doped mesoporous carbon as metal-free electrocatalysts for the *in-situ* production of hydrogen peroxide. Carbon 95, 949–963. 10.1016/j.carbon.2015.09.002

[B26] Shen B. Lu D. Zhai W. Zheng W. (2013). Synthesis of graphene by low-temperature exfoliation and reduction of graphite oxide under ambient atmosphere. J. Mater. Chem. C 1, 50–53. 10.1039/C2TC00044J

[B27] Sheng Z. H. Shao L. Chen J. J. Bao W. J. Wang F. B. Xia X. H. (2011). Catalyst-free synthesis of nitrogen-doped graphene via thermal annealing graphite oxide with melamine and its excellent electrocatalysis. ACS Nano 5, 4350–4358. 10.1021/nn103584t 21574601

[B28] Singh D. K. Jenjeti R. N. Eswaramoorthy S. S. M. (2017). Two in one: N-doped tubular carbon nanostructure as an efficient metal-free dual electrocatalyst for hydrogen evolution and oxygen reduction reactions. J. Mater. Chem. A 5, 6025–6031. 10.1039/C6TA11057F

[B29] Sun M. Wu X. B. Xie Z. Y. Deng X. T. Wen J. Y. Huang Q. Z. (2017). Tailoring platelet carbon nanofibers for high-purity Pyridinic-N doping: a novel method for synthesizing oxygen reduction reaction catalysts. Carbon 125, 401–408. 10.1016/j.carbon.2017.09.085

[B30] Sun T. Wang J. Qiu C. T. Ling X. Tian B. B. Chen W.. (2018). B, N codoped and defect-rich nanocarbon material as a metal-free bifunctional electrocatalyst for oxygen reduction and evolution reactions. Adv. Sci. 5:1800036. 10.1002/advs.201800036 30027038 PMC6051395

[B31] Tian J. Q. Liu Q. Asiri A. M. Alamry K. A. Sun X. P. (2014). Ultrathin graphitic C_3_N_4_ nanosheets/graphene composites: efficient organic electrocatalyst for oxygen evolution reaction. ChemSusChem 7, 2125–2132. 10.1002/cssc.201402118 24823866

[B32] Wang C. H. Hu F. Yang H. C. Zhang Y. J. Lu H. Wang Q. B. (2017a). 1.82 wt.% Pt/N, P co-doped carbon overwhelms 20 wt.% Pt/C as a high-efficiency electrocatalyst for hydrogen evolution reaction. Nano Res. 10, 238–246. 10.1007/s12274-016-1281-9

[B33] Wang N. Li T. F. Song Y. Liu J. J. Wang F. (2017b). Metal-free nitrogen-doped porous carbons derived from pomelo peel treated by hypersaline environments for oxygen reduction reaction. Carbon 130, 692–700. 10.1016/j.carbon.2018.01.068

[B34] Wu Z. S. Ren W. Gao L. Liu B. Jiang C. Cheng H. M. (2009). Synthesis of high-quality graphene with a pre-determined number of layers. Carbon 7, 493–499. 10.1016/j.carbon.2008.10.031

[B35] Wu Z. X. Wang J. Song M. Zhao G. M. Zhu Y. Fu G. T.. (2018). Boosting oxygen reduction catalysis with N-doped carbon coated Co_9_S_8_ microtubes. ACS Appl. Mater. Interfaces 10, 25415–25421. 10.1021/acsami.8b07207 29979562

[B36] Xiao J. Zhang Z. Y. Zhang Y. Lv Q. Y. Jing F. Chi K. (2018). Large-scale printing synthesis of transition metal phosphides encapsulated in N, P co-doped carbon as highly efficient hydrogen evolution cathodes. Nano Energy 51, 223–230. 10.1016/j.nanoen.2018.06.040

[B37] Xie L. J. Sun G. H. Su F. Y. Guo X. Q. Kong Q. Q. Li X. M. (2016). Hierarchical porous carbon microtubes derived from willow catkins for supercapacitor applications. J. Mater. Chem. A 4, 1637–1646. 10.1039/C5TA09043A

[B38] Xu Z. Q. Ma J. H. Shi M. H. Xie Y. H. Feng C. (2018). Biomass based iron and nitrogen co-doped 3D porous carbon as an efficient oxygen reduction catalyst. J. Colloid Interface Sci. 523, 144–150. 10.1016/j.jcis.2018.03.092 29614423

[B39] Yang M. J. Wu D. F. Cheng D. J. (2019). Biomass-derived porous carbon supported Co-CoO yolk-shell nanoparticles as enhanced multifunctional electrocatalysts. Int. J. Hydrogen Energy 44, 6525–6534. 10.1016/j.ijhydene.2019.01.155

[B40] Yu W. H. Wang H. L. Liu S. Mao N. Liu X. Shi J. (2016). N, O-codoped hierarchical porous carbons derived from algae for high-capacity supercapacitors and battery anodes. J. Mater. Chem. A 4, 5973–5983. 10.1039/C6TA01821A

[B41] Zeng D. R. Yu X. Zhan Y. F. Cao L. M. Wu X. X. Zhang B. D. (2016). Insight into the nitrogen-doped carbon as oxygen reduction reaction catalyst: the choice of carbon/nitrogen source and active sites. Int J Hydrogen Energy 41, 8563–8575. 10.1016/j.ijhydene.2016.03.072

[B42] Zhan T. R. Lu S. S. Liu X. L. Teng H. N. Hou W. G. (2018). Alginate derived Co_3_O_4_/Co nanoparticles decorated in N-doped porous carbon as an efficient bifunctional catalyst for oxygen evolution and reduction reactions. Electrochim. Acta 265, 681–689. 10.1016/j.electacta.2018.02.006

[B43] Zhang C. L. Wang B. W. Shen X. C. Liu J. W. Kong X. K. Chuang S. S. C. (2016b). A nitrogen-doped ordered mesoporous carbon/graphene framework as bifunctional electrocatalyst for oxygen reduction and evolution reactions. Nano Energy 30, 503–510. 10.1016/j.nanoen.2016.10.051

[B44] Zhang S. L. Huang W. Hu P. Huang C. Shang C. Zhang C. (2015). Conjugated microporous polymers with excellent electrochemical performance for lithium and sodium storage. J. Mater. Chem. A 3, 1896–1901. 10.1039/C4TA06058J

[B45] Zhang Z. P. Qin Y. S. Dou M. L. Ji J. Wang F. (2016a). One-step conversion from Ni/Fe polyphthalocyanine to N-doped carbon supported Ni-Fe nanoparticles for highly efficient water splitting. Nano Energy 30, 426–433. 10.1016/j.nanoen.2016.10.035

[B46] Zhao Y. F. Ran W. He J. Song Y. F. Zhang C. M. Xiong D. B.. (2015). Oxygen-rich hierarchical porous carbon derived from artemia cyst shells with superior electrochemical performance. ACS Appl. Mater. Interfaces 7, 1132–1139. 10.1021/am506815f 25531022

[B47] Zhou H. Zhang J. Amiinu I. S. Zhang C. Y. Liu X. B. Tu W. M.. (2016). Transforming waste biomass with an intrinsically porous network structure into porous nitrogen doped graphene for highly efficient oxygen reduction. Phys. Chem. Chem. Phys. 18, 10392–10399. 10.1039/C6CP00174B 27030144

[B48] Zhou Q. X. Su Z. B. Tang Y. D. Ai L. Fu G. T. Wu Z. X.. (2019). Pt-like oxygen reduction activity induced by cost-effective MnFeO_2_/N-Carbon. Chem.-Eur. J. 25, 6226–6232. 10.1002/chem.201900638 30860616

[B49] Zhuang L. Z. Jia Y. He T. W. Du A. J. Yan X. C. Ge L. (2018). Tuning oxygen vacancies in two-dimensional iron-cobalt oxide nanosheets through hydrogenation for enhanced oxygen evolution activity. Nano Res. 11, 3509–3518. 10.1007/s12274-018-2050-8

[B50] Zuo L. X. Wang W. J. Song R. B. Lv J. J. Jiang L. P. Zhu J. J. (2017). NaCl crystal tuning nitrogen self-doped porous graphitic carbon nanosheets for efficient oxygen reduction. ACS Sust. Chem. Eng. 5, 10275–10282. 10.1021/acssuschemeng.7b02291

