# Supplementary material for: Catalytically Active Carbon From Cattail Fibers for Electrochemical Reduction Reaction
Source: Front Chem. 2019 Nov 19;7:786. doi: 10.3389/fchem.2019.00786 (PMC6878766; doi:10.3389/fchem.2019.00786)
Supplement: Supplementary file 1 [file Table_1.DOC]

## Supplementary Materials (SM) for:

## Catalytically Active Carbon from Cattail Fibers for Electrochemical Reduction Reaction

Yanyan Liu,*^,†, ‡^ Meifang Hu,^‡^ Wei Xu,^†^ Xianli Wu,^‡^ and Jianchun Jiang*^,†^

^†^ Institute of Chemical Industry of Forest Products, CAF; National Engineering Lab. for Biomass Chemical Utilization; Key Lab. of Chemical Engineering of Forest Products, National Forestry and Grassland Administration; Key Lab. of Biomass Energy and Material, Jiangsu Province; Co-Innovation Center of Efficient Processing and Utilization of Forest Resources, Nanjing 210042, P R China

^‡^ College of Chemistry, Zhengzhou University, 100 Science Road, Zhengzhou 450001, P. R. China

* Corresponding Author. E-mail: lyylhs180208@163.com (Y.Y. Liu), and jiangjc@icifp.cn (J.C. Jiang).

**Total number of pages: 6**

**Total number of Figures: 4**

**Total number of table: 3**

**Table of Contents**

Figure S1……………………………………………………………………………...……...S2

Figure S2…………………………………………………………………………….….....…S3

Figure S3……………………………………………………………………………...……...S5

Figure S4…………………………………………………………………………….….....…S6

Table S1…………………………………………………………………………….….........S4

Table S2…………………………………………………………………………….….........S4

Table S3…………………………………………………………………………….….........S4

Table S4…………………………………………………………………………….….........S7

Table S5…………………………………………………………………………….….........S7

Reference…………………………………………………………………………….….........S8

The main possible reactions between KHCO_3_ and C are as follows:

2KHCO_3_→K_2_CO_3_+CO_2_+H_2_O

K_2_CO_3_→K_2_O+CO_2_

K_2_CO_3_+2C→2K+3CO

K_2_O+C→2K+CO

CO_2_+C→2CO


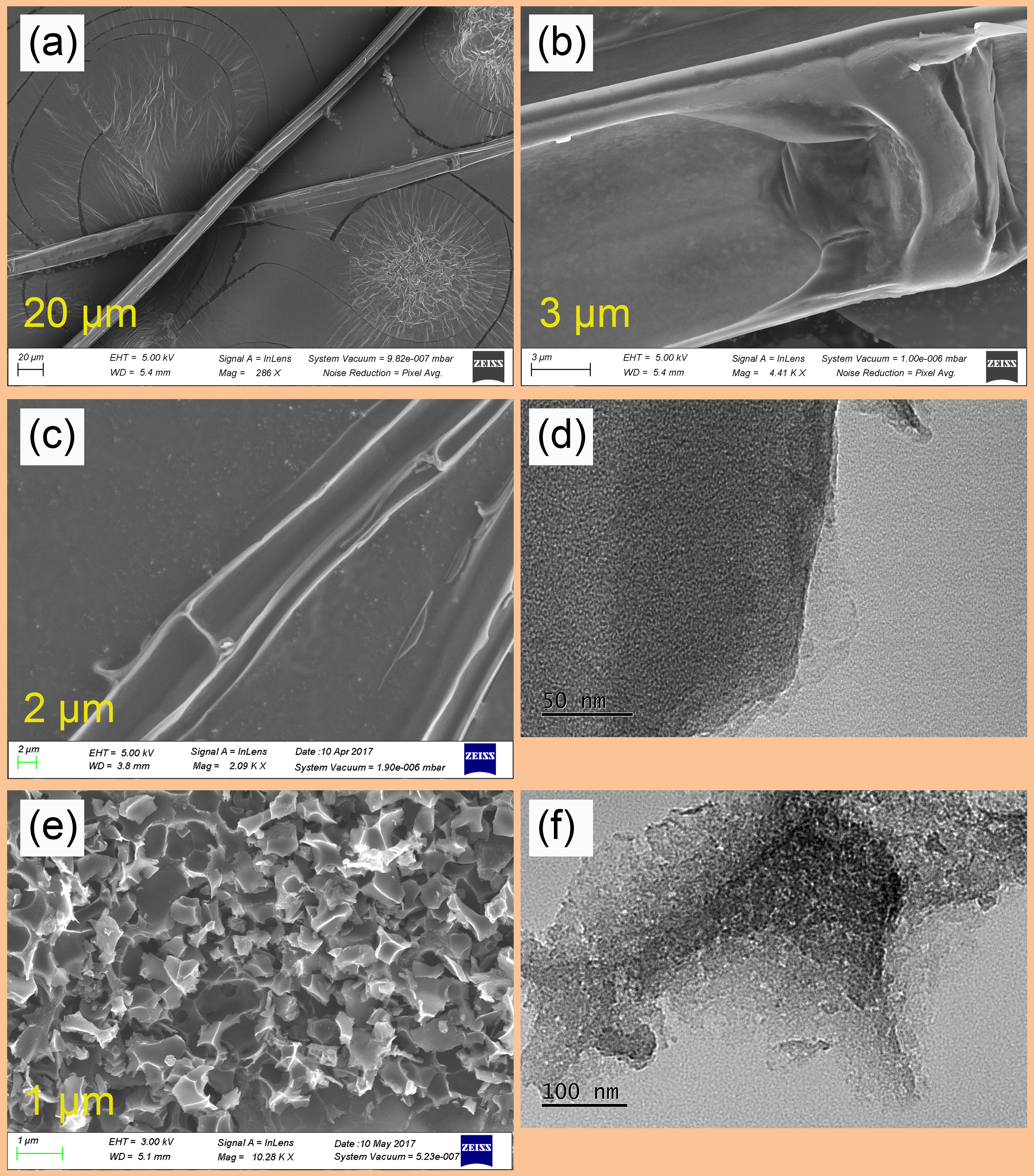


**Figure S1.** (a, b) The SEM images of cattail fibers, the SEM image and the TEM image of (c, d) NCF and (e, f) HPCF.

**
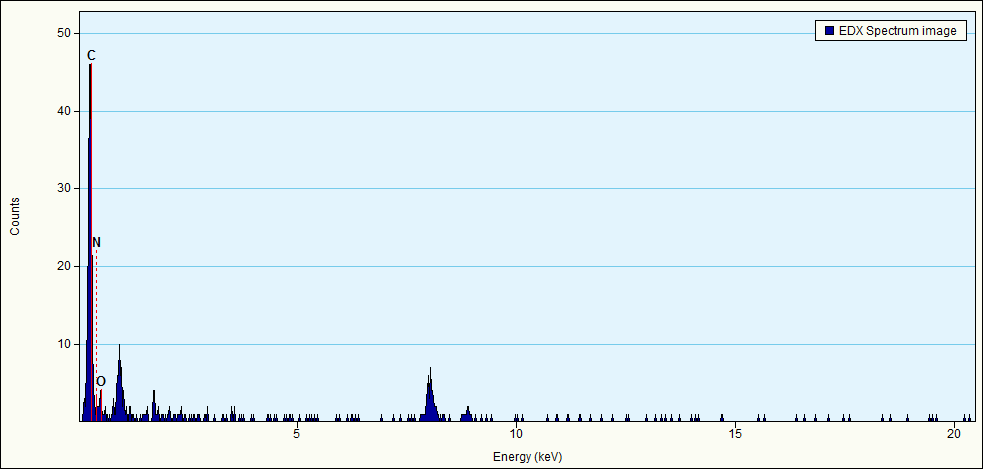
**

**Figure S2.** The EDX spectrum image of NHPCF.

**Table S1.** The atomic ratios of various catalysts from elemental analysis.

| **Materials** | **C** | **N** | **S** | **H** |
| --- | --- | --- | --- | --- |
|  | Atomic ratio (atm.%) | | | |
| NCF  HPCF | 78.0  72.0 | 5.48  0.49 | 2.40 | 1.46  1.5 |
| NHPCF | 88.6 | 6.35 | 0.85 | 1.4 |

**Table S2.** The elemental ratios of various materials from XPS spectra.

| Elements | C1s | N1s | O1s | Cl2p |
| --- | --- | --- | --- | --- |
|  | Atomic ratio (atm.%) | | | |
| HPCF | 90.57 | 0.62 | 7.36 | 0.18 |
| NHPCF | 82.12 | 7.43 | 10.46 | 0.00 |

**Table S3.** The *t*-Plot report of NCF, HPCF and NHPCF.

| *t*-Plot report | *S*_micro_ | *S*_external_ | *V*_micro_ | *V*_total_ |
| --- | --- | --- | --- | --- |
|  | m^2^·g^−1^ | | cm^3^·g^−1^ | |
| NCF | 555 | 67 | 0.212 | 0.266 |
| HPCF | 626 | 1712 | 0.297 | 1.418 |
| NHPCF | 572 | 1773 | 0.277 | 1.416 |


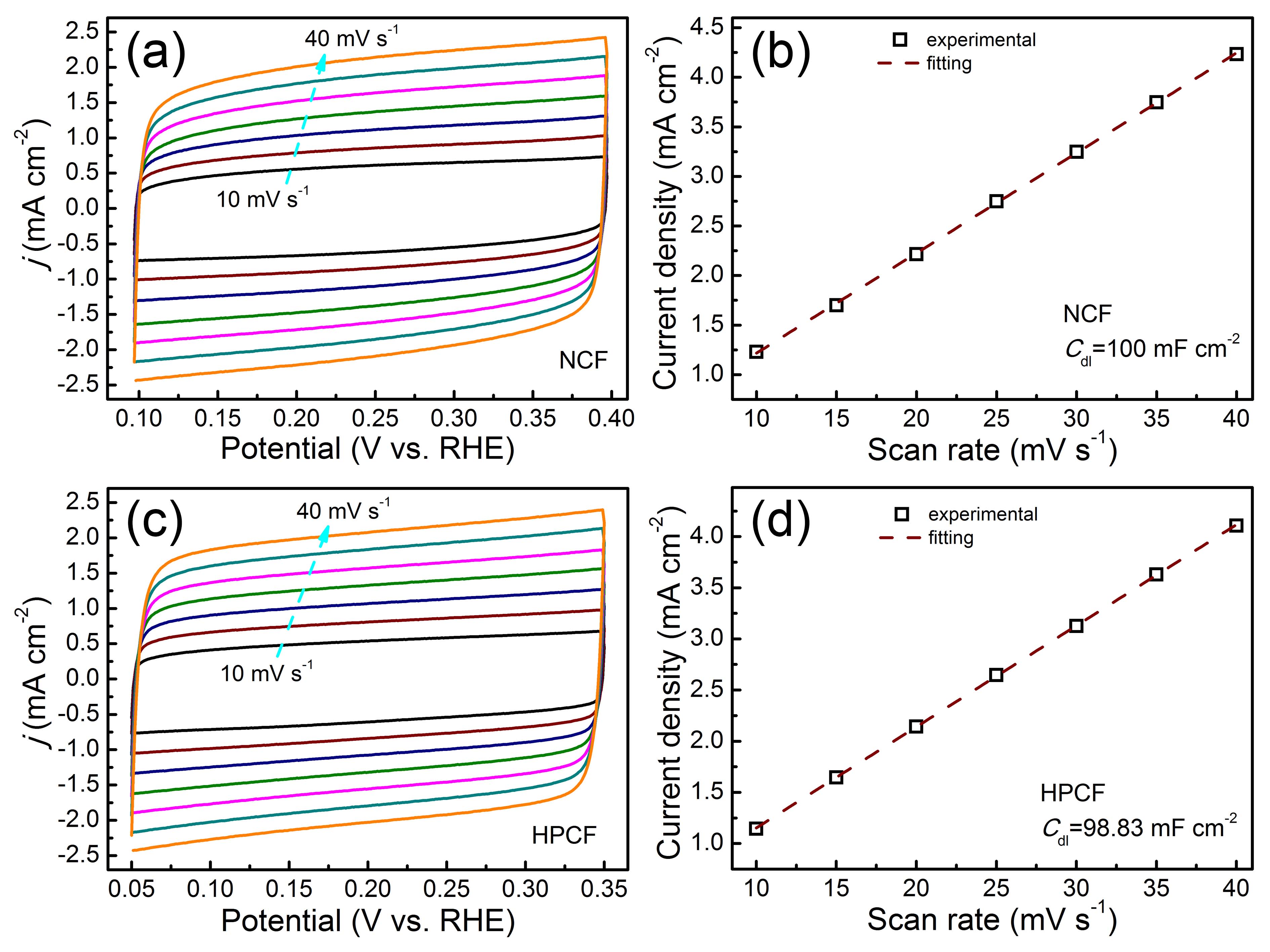


**Figure S3.** (a, c) CV curves of NCF and NHPCF at different scan rates, (b, d) corresponding evaluation of the *C*_dl_ for NCF and NHPCF, respectively.


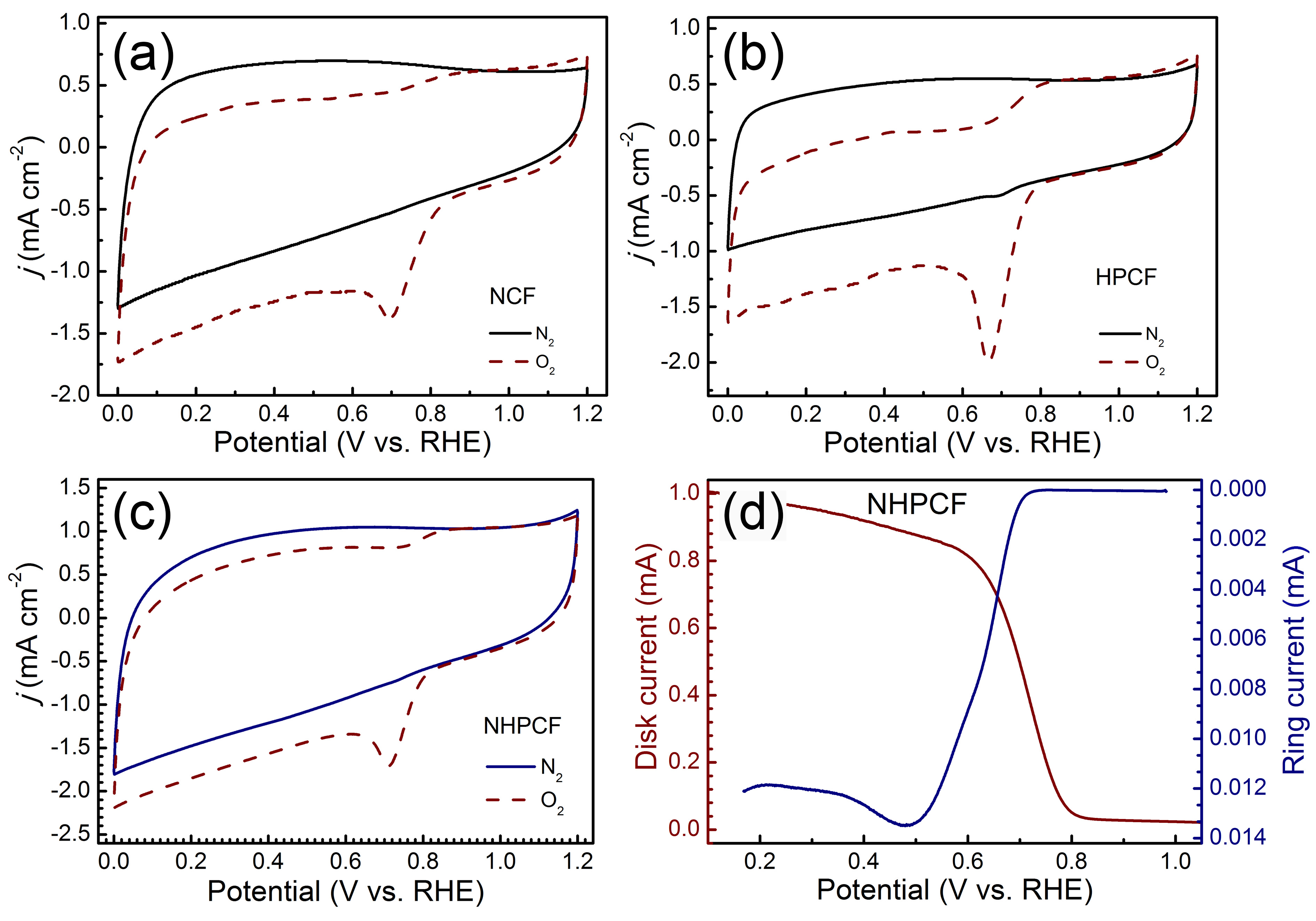


**Figure S4.** (a-c) CV curves of NCF, HPCF and NHPCF, and (d) RRDE voltammograms of NHPCF.

**Table S4.** The HER catalytic activity of some reported carbon-based electrocatalysts.

| **Catalyst** | Current density (mA cm^-2^) | Overpotential | Tafel slope | Reference |
| --- | --- | --- | --- | --- |
| NDC-800 | 10 | 276 | 94 | S1 |
| N,P co-doped graphene | 10 | 420 | 91 | S2 |
| NPCF | 10 | 244 | 135 | S3 |
| Co-NRCNTs | 10 | 260 | 80 | S4 |
| N,S co-doped graphene | 10 | 276 | 81 | S5 |
| CoO@Co/N-rGO | 10 | 146 | 67 | S6 |
| C−N doped MoS_2_/C catalyst | 10 | 167 | 56 | S7 |
| CoS_2_/CC | 10 | 288 | 210.7 | S8 |
| MoS_x_/3D-graphene | 10 | 163 | 41 | S9 |
| NHPCF | 10 | 150 | 89 | In this work |

**Table S5.** The ORR catalytic activity of some reported carbon-based electrocatalysts.

| **Catalyst** | *E*_onset_ [V]  (V VS. RHE) | *E_1/2_* [V]  (V VS. RHE) | Transferred electrons (*n*) | Reference |
| --- | --- | --- | --- | --- |
| NCS-800 | - | 0.75 | 3.7–4.0 | S10 |
| NPBC-2 | 0.90 | 0.75 | 3.78–3.90 | S11 |
| N-C@CNT-900 | 0.94 | 0.81 | 3.9 | S12 |
| SAR-800 | - | 0.79 | 4 | S13 |
| N-CG–CoO | 0.90 | 0.81 | 4 | S14 |
| Co_3_O_4_-N-rmGO | - | 0.83 | 3.6 | S15 |
| Fe-N/C-800 | 0.92 | 0.81 | 4 | S16 |
| GluSo_1000 | 0.90 | 0.76 | 3.51–3.82 | S17 |
| AC-F-U-P | 0.94 | 0.75 | 3.7 | S18 |
| NHPCF | 0.92 | 0.82 | 3.9 | In this work |

**Reference**

S1 Singh, D.K., Jenjeti, R.N., Sampathb, S., Eswaramoorthy, M. (2017). Two in one: N-doped tubular carbon nanostructure as an efficient metal-free dual electrocatalyst for hydrogen evolution and oxygen reduction reactions. *J. Mater. Chem. A* 5, 6025–6031. doi: 10.1039/C6TA11057F

S2 Zheng, Y., Jiao, Y., Jaroniec, M., Qiao, S.Z. (2015). Advancing the electrochemistry of the hydrogen‐evolution reaction through combining experiment and theory. *Angew. Chem. Int. Ed.* 54, 52–65. doi: 10.1002/anie.201407031

S3 Han, G.S., Hu, M.F., Liu, Y.Y., Gao, J., Han, L., Lu, S.Y., et al. (2019). Efficient carbon-based catalyst derived from natural cattail fiber for hydrogen evolution reaction. *J. Solid State Chem.* 274, 207–214. doi: 10.1016/j.jssc.2019.03.027

S4 Zou, X., Huang, X., Goswami, A., Silva, R., Sathe, B.R., Mikmeková, E., et al. (2014). Cobalt‐embedded nitrogen‐rich carbon nanotubes efficiently catalyze hydrogen evolution reaction at all pH values. *Angew. Chem. Int. Ed.* 53, 4372-4376. doi: 10.1002/anie.201311111

S5 Ito, Y., Cong, W.T., Fujita, T., Tang, Z., Chen, M.W. (2014). High catalytic activity of nitrogen and sulfur co-doped nanoporous graphene in the hydrogen evolution reaction. *Angew. Chem. Int. Ed.* 54, 2131–2136. doi:10.1002/anie.201410050

S6 Liu, X.X., Zang, J.B., Chen, L., Chen, L.B., Chen, X., Wu, P., et al. (2017). A microwave-assisted synthesis of CoO@Co core–shell structures coupled with N-doped reduced graphene oxide used as a superior multi-functional electrocatalyst for hydrogen evolution, oxygen reduction and oxygen evolution reactions. *J. Mater. Chem. A* 5, 5865-5872. doi: 10.1039/c6ta10591b

S7 Cai, W.W., Luo, X.Y., Jiang, Y., Liu, Z., Li, J., Ma, L., et al. (2018). Nitrogen doped carbon active sites boost the ultra-stable hydrogen evolution reaction on defect-rich MoS_2_ nanosheets. *Int. J. Hydrogen Energy* 43, 2026-2033. doi: 10.1016/j.ijhydene.2017.12.059

S8 Huang, J., Hou, D., Zhou, Y., Zhou, W., Li, G., Tang, Z., et al. (2015). MoS_2_ nanosheet-coated CoS_2_ nanowire arrays on carbon cloth as three-dimensional electrodes for efficient electrocatalytic hydrogen evolution. *J. Mater. Chem. A* 3, 22886-22891. doi: 10.1039/c5ta07234d

S9 Hung, Y.-H., Su, C.-Y. (2017). Highly efficient electrocatalytic hydrogen production via MoSx/3D-graphene as hybrid electrode. *Int. J. Hydrogen Energy* 42(34), 22091-22099. doi:10.1016/j.ijhydene.2017.04.199

S10 Chen, P., Wang, L.-K., Wang, G., Gao, M.-R., Ge, J., Yuan, W.-J., Yu, S.-H. (2014). Nitrogen-doped nanoporous carbon nanosheets derived from plant biomass: an efficient catalyst for oxygen reduction reaction. *Energy Environ. Sci.* 7(12), 4095-4103. doi:10.1039/c4ee02531h

S11 Zheng, X., Cao, X., Wu, J., Tian, J., Jin, C., Yang, R. (2016). Yolk-shell N/P/B ternary-doped biocarbon derived from yeast cells for enhanced oxygen reduction reaction. *Carbon* 107, 907-916. doi:10.1016/j.carbon.2016.06.102

S12 Guo, C.Z., Liao, W.L., Li, Z.B., Sun, L.T., Chen, C.G. (2013). Easy conversion of protein-rich enoki mushroom biomass to nitrogen-doped carbon nanomaterial as a promising metal-free catalyst for oxygen reduction reaction. *Nanoscale* 7, 15990-15998. doi: 10.1039/c5nr03828f

S13 Mondal, D., Sharma, M., Wang, C.H., Lin, Y.C., Huang, H.C., Saha, A., et al. (2016). Deep eutectic solvent promoted one step sustainable conversion of fresh seaweed biomass to functionalized graphene as a potential electrocatalyst. *Green Chem.* 18, 2819-2826. doi: 10.1039/c5gc03106k

S14 Mao, S., Wen, Z.H., Huang, T.Z., Hou, Y., Chen, J.H. (2014). High-performance bi-functional electrocatalysts of 3D crumpled graphene–cobalt oxide nanohybrids for oxygen reduction and evolution reactions. *Energy Environ. Sci.* 7, 609-616. doi: 10.1039/c3ee42696c

S15 Cao, B.F., Veith, G.M., Diaz, R.E., Liu, J., Stach, E.A., Adzic, R.R., Khalifah, P.G. (2013). Cobalt molybdenum oxynitrides: synthesis, structural characterization, and catalytic activity for the oxygen reduction reaction. *Angew. Chem. Int. Ed.* 52, 10753-10757. doi: 10.1002/anie.201303197

S16 Lin, L., Zhu, Q., Xu, A.W. (2014). Noble-Metal-Free Fe–N/C Catalyst for Highly Efficient Oxygen Reduction Reaction under Both Alkaline and Acidic Conditions. *J. Am. Chem. Soc.* 136, 11027-11033. doi: org/10.1021/ja504696r

S17 Alatalo, S.M., Qiu, K.P., Preuss, K., Marinovic, A., Sevilla, M., Sillanpaa, M., et al. (2015). Soy protein directed hydrothermal synthesis of porous carbon aerogels for electrocatalytic oxygen reduction. *Carbon* 96, 622–630. doi: 10.1016/j.carbon.2015.09.108

S18 Boighei, M., Laocharoen, N., Kibena-Poldsepp, E., Johansson, L.S., Campbell, J., Kauppinen, E., et al. (2017). Porous N,P-doped carbon from coconut shells with high electrocatalytic activity for oxygen reduction: Alternative to Pt-C for alkaline fuel cells. *Appl. Catal. B* 204, 394–402. doi: org/10.1016/j.apcatb.2016.11.029
